# Mesolimbic dopamine neurons drive infradian rhythms in sleep-wake and heightened activity state

**DOI:** 10.1126/sciadv.ado9965

**Published:** 2025-01-01

**Authors:** Pratap S. Markam, Clément Bourguignon, Lei Zhu, Bridget Ward, Martin Darvas, Paul V. Sabatini, Maia V. Kokoeva, Bruno Giros, Kai-Florian Storch

**Affiliations:** ^1^ Integrated Program in Neuroscience, McGill University, Montreal, QC H3A 2B4, Canada.; ^2^ Douglas Mental Health University Institute, McGill University, Montreal, QC H4H 1R3, Canada.; ^3^ Department of Laboratory Medicine and Pathology, University of Washington, Seattle, WA 98195, USA.; ^4^ Division of Endocrinology and Metabolism, Department of Medicine, McGill University, Montreal, QC H3A 2B4, Canada.; ^5^ Department of Psychiatry, McGill University, Montreal, QC H4H 1R3, Canada.; ^6^ Université de Paris Cité, INCC UMR 8002, CNRS, F-75006 Paris, France.

## Abstract

Infradian mood and sleep-wake rhythms with periods of 48 hours and beyond have been observed in patients with bipolar disorder (BD), which even persist in the absence of exogenous timing cues, indicating an endogenous origin. Here, we show that mice exposed to methamphetamine in drinking water develop infradian locomotor rhythms with periods of 48 hours and beyond which extend to sleep length and manic state–associated behaviors in support of a model for cycling in BD. The cycling capacity is abrogated upon genetic disruption of dopamine (DA) production in DA neurons of the ventral tegmental area (VTA) or ablation of nucleus accumbens projecting DA neurons. Furthermore, chemogenetic activation of ^VTA^DA neurons including those that project to the nucleus accumbens led to locomotor period lengthening in circadian clock–deficient mice, which was counteracted by antipsychotic treatment. Together, our findings argue that BD cycling relies on infradian rhythm generation that depends on mesolimbic DA neurons.

## Teaser

Ablation of the dopamine neurons of the ventral tegmental area abolishes the capacity to produce infradian behavioral rhythms.

## INTRODUCTION

Sleep-wake rhythms are typically aligned with the solar day, adhering to a periodicity of 24 hours. However, there are also accounts of sleep-wake cycling with periods longer than a day. Individuals affected by “non–24-hour sleep-wake rhythm disorder” (N24SWD) (*1*) exhibit sleep-wake rhythms that can reach far into the infradian (≫24 hours) period range (*2*). Infradian sleep-wake cycles with a period of 48 hours have been specifically reported in the context of bipolar disorder (BD) (*3*–*5*). Here, sleep length varies between successive days, and this sleep cycling typically goes hand in hand with mood states alternating between (hypo)mania on short-sleep days and depression on long-sleep days. BD 48-hour cycling persists even under conditions of time isolation, i.e., in the absence of any exogenous timing cues (*5*), indicating an endogenous origin; however, as with infradian N24SWD, the biological basis of BD-associated 48-hour cycling is currently unknown.

Sleep-wake rhythms longer than 24 hours can be experimentally induced in laboratory animals upon chronic treatment with methamphetamine (Meth) via drinking water (*6*, *7*). Upon Meth exposure, a second rhythmic component (2ndC) emerges alongside the circadian rhythm, which develops over the course of several days to weeks and whose period is affected by the concentration of Meth. This 2ndC can be produced even in the absence of the circadian timer, underscoring its independence from the canonical clock machinery (*8*, *9*). A 2ndC can also emerge upon constant Meth infusion via implanted mini pumps (*8*), arguing against rhythmic Meth uptake through intermittent drinking as driver of the 2ndC. A key target of Meth is the dopamine (DA) transporter (DAT), which is set into inverse action upon Meth exposure, turning it into a DA releasing entity, thereby elevating extracellular DA (*10*). Consistently, *DAT* gene disruption, which also causes an increase in extracellular DA (*11*), equally leads to the emergence of a 2ndC (*12*), a finding that also demonstrates that 2ndC generation does not solely rely on Meth treatment. Together, these data suggest that DA neurons, which are the near-exclusive sites of DAT expression, exert a crucial role in the genesis of a second rhythmic locomotor component (*12*), possibly harboring a dopamine-based oscillator (DO) that drives it. Here, we set out to identify the neuronal substrate of the oscillator and to find evidence in mice for its role as the driver of behavioral cycling in BD.

## RESULTS

### Meth-induced rhythms can reach far into the infradian range

**Fig. 1.**
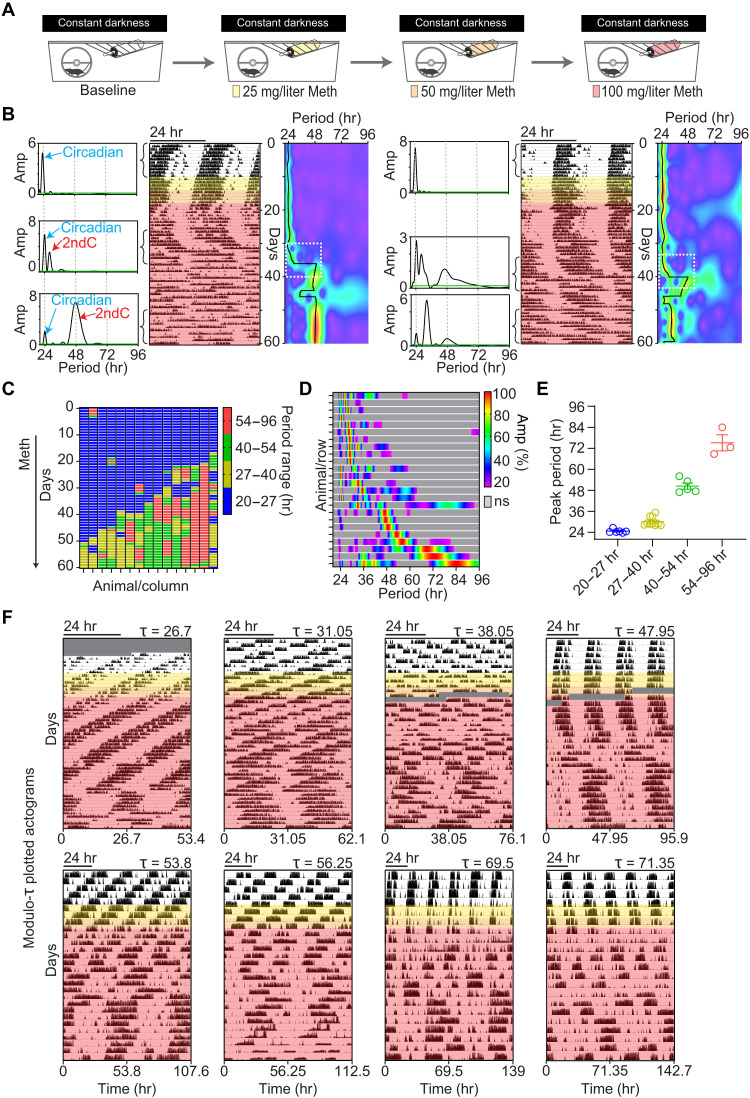
Variability and extent of infradian rhythm induction by Meth. (**A**) Experimental regimen of running wheel activity monitoring in response to escalating concentrations of Meth in drinking water. (**B**) Representative actograms displaying running wheel activity patterns of two single-housed animals exposed to Meth. Meth concentrations applied are indicated by color shading in correspondence to (A). Lomb-Scargle periodograms on the left and CWT heatmaps on the right of each actogram show the emergence and evolution of infradian locomotor rhythms. Green line in periodograms demarcates the significant threshold for rhythmicity. Black traces in heatmaps are the wavelet ridges reporting the instantaneous peak period. hr, hours; ns, not significant. (**C**) Evolution of the peak period across animals identified by the wavelet ridge of the CWT. Time course of Meth treatment as indicated in (B). (**D**) Composite heatmap of normalized periodograms from the final 14 days of Meth treatment suggests profound period flexibility. (**E**) Binned display of highest peaks derived from periodograms shown in (D). Shown are means ± SEM. (**F**) Representative actograms of Meth-treated mice plotted modulo according the peak period from the last 14 days of the 60-day actogram. The modulo actograms demonstrate that mice can adopt robust rhythmicity in the near to far infradian range upon Meth exposure via drinking water. Grayed areas indicated data loss.

Now, Meth-induced 2ndCs are generally viewed as additional circadian rhythms as they typically exhibit periods not far from 24 hours. However, in most of the respective studies, Meth has been used at concentrations up to 50 mg/liter of drinking water, which may account for the rather limited period range that was observed (*13*). Here, we examined running wheel activity of mice exposed to increasing levels of Meth, up to 100 mg/liter, which is twice the concentration normally used (Fig. 1A). As expected, Meth treatment led to the emergence of a 2ndC over time demonstrated by the pattern of running wheel activity and associated periodogram analysis (Fig. 1B). However, time of 2ndC emergence with respect to Meth treatment onset was found to be variable, with some animals readily showing a 2ndC, while others took weeks to respond (Fig. 1C). There was also considerable variability in the period that the 2ndC adopts in response to Meth, with faster responding animals leaning toward longer-period 2ndCs (Fig. 1C). In general, 2ndC emergence is preceded by a gradual lengthening of the main (circadian) locomotor component [Fig. 1B, dashed white box in continuous wavelet transform (CWT) displays right next to actograms). Confirming previous findings (*14*–*16*), the 2ndC can gradually or abruptly change its period over time (Fig. 1, B and C) which can be viewed as reflections of a rather labile oscillator that drives the 2ndC. Figure 1B (actogram on the right) further demonstrates another known feature of the second component: relative coordination with the circadian timer, i.e., the period of the 2ndC changes depending on its phase angle position relative to the circadian timer (*6*, *7*). Upon prolonged administration of Meth (100 mg/liter), we found a high fraction of animals to exhibit 2ndCs with periods in the far infradian range (Fig. 1, C to F), with a considerable portion (≥30%) reaching periods beyond 40 hours (Fig. 1E). The relatively even distribution of infradian peaks across a wide infradian range demonstrated by the heatmap display of individual periodograms from animals under Meth treatment (Fig. 1D) underscores a profound period flexibility. This is reflective of a highly tunable rhythm generator (*12*), which is in stark contrast to the circadian oscillator whose period range is rather constrained to near 24-hour periodicities. Figure 1F shows actograms plotted in modulo according to the ultimate period that the animals adopt, illustrating the regularity and robustness of Meth-induced infradian sleep-wake rhythms. Together, we show that chronic Meth treatment leads to the emergence of a rhythmic locomotor component of varying periodicities that often reaches far into the infradian range (>40 hours), arguing that the oscillator that drives the 2ndC is not circadian in nature as previously suggested (*7*). Notably, we did not find any overt sex differences in 2ndC induction. The time to infradian rhythm emergence was indifferent between males and females as was the peak period reached after 9 weeks of Meth (fig. S1).

### Infradian rhythmic mice model aspects of bipolar cycling

**Fig. 2.**
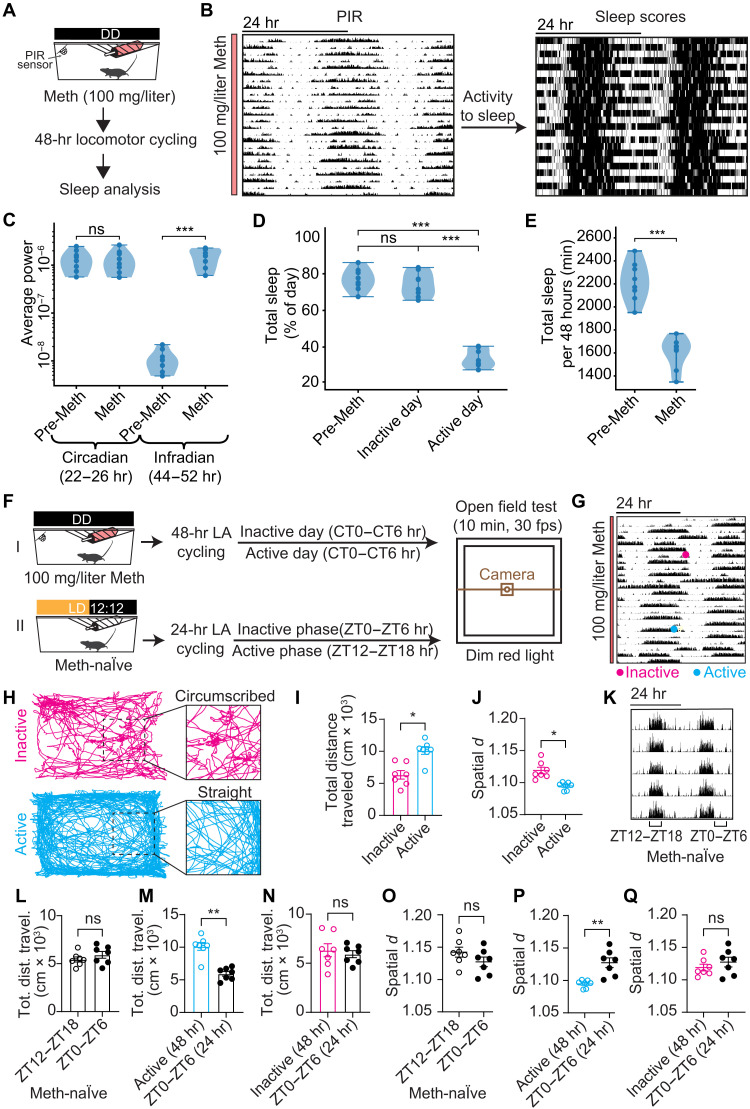
Forty-eight–hour rhythmic mice show cycling in sleep and mania-associated behaviors. (**A**) Regimen to record sleep during 48-hour rhythmicity. DD, constant darkness. (**B**) PIR-derived spontaneous locomotor activity (left) and sleep (right) of a 48-hour rhythmic mouse. (**C**) Rhythmic strength (scale-averaged spectral power) in the circadian (22- to 26-hour) and infradian (44- to 52-hour) range before Meth exposure and during 48-hour cycling. (**D**) Daily total sleep before Meth and on active and inactive days while cycling. (**E**) Daily sleep (average across four consecutive days) before Meth and during 48-hour cycling. (**F**) Experimental design for OFT in 48- and 24-hour rhythmic mice. fps, frames per second; LD, light-dark cycle; LA, locomotor activity. (**G** and **H**) Actogram (G) of a 48-hour cycling mouse used for OFT analysis. Time of testing during active/inactive days is indicated. (H) Representative locomotor traces derived from 10-min open field video recording on inactive and active days, as indicated in (G). (**I**) Total distance traveled during OFT of 48-hour cycling mice during their active and inactive days. (**J**) Spatial *d* in 48-hour cycling mice during inactive and active days. (**K**) Representative actogram of Meth-naïve animals displaying 24-hour rhythms in locomotor activity. (**L**) Total distance traveled during OFT conducted during the active (ZT12 to ZT18) and inactive (ZT0 to ZT6) phases of 24-hour rhythmic, Meth-naïve mice. (**M** and **N**) Total distance traveled on active (M) and inactive (N) days by 48-hour cyclers compared to the inactive phase of Meth-naïve mice. (**O**) Spatial *d* in Meth-naïve mice during their active versus inactive phase. (**P** and **Q**) Spatial *d* on active (P) and inactive (Q) days by 48-hour cyclers compared to the inactive phase of Meth-naïve mice. Means ± SEM. *n* = 8 [(C) to (E)] and 7 [(I), (J), and (L) to (Q)]. Mann-Whitney test, (C) to (E) and (L) to (Q); Wilcoxon’s test, (I) and (J). *P < 0.05; **P < 0.01; ***P < 0.001.

To demonstrate that similar to patients with BD with 48-hour cycling (*3*–*5*), the 48-hour rhythmicity in mice also extends to sleep, we examined the effect of chronic Meth treatment on spontaneous locomotor activity (LA) recorded by passive infrared sensors (PIRs) (Fig. 2A). Using this approach, we found that even in the absence of a running wheel, mice consistently adopted 48-hour rhythmicity in locomotor activity in response to Meth (Fig. 2B and fig. S2A). We then transformed the PIR data into sleep scores in accordance with a procedure that has been validated against electroencephalography-derived sleep (*17*, *18*). The resulting sleep pattern (Fig. 2B, right) showed a 48-hour rhythmicity expectedly mirroring the PIR-derived activity pattern (Fig. 2B, left). Figure 2C confirms that infradian spectral power in sleep rhythmicity is virtually absent before Meth exposure but profoundly present when mice exhibited 48-hour cycling (see also fig. S2, B and C). When comparing successive days during 48-hour cycling, we found sleep length to differ strongly. On active days, mice spent 30 to 40% of the day sleeping, whereas they allocated 60 to 80% of the day to sleep on inactive days or days before Meth treatment (Fig. 2D), which agrees well with observations in patients with BD with 48-hour cycling who alternate between short sleep and a rather standard sleep length (*3*, *5*). Consistently, total sleep during 48-hour cycling was lower when compared to pre-Meth conditions (Fig. 2E), in support of a substantially reduced sleep need in 48-hour cycling mice or humans.

To further corroborate the validity of 48-hour rhythmic mice as model for 48-hour cycling in BD, we assessed whether these mice show evidence of hyperactivity every other day using the open field test (OFT). As hyperactivity or psychomotor agitation is a hallmark of BD mania (*19*), such a finding would be consistent with these mice entering a mania-like state every other day. Open field video tracking revealed that distance traveled over time was elevated when 48-hour cycling animals were tested on days when they slept little (active days) compared to the alternating days of long sleep (inactive days) (Fig. 2, F to I). In contrast, no difference was found in Meth-naïve animals, when tested during their corresponding active [zeitgeber time 12 (ZT12) to ZT18] and inactive (ZT0 to ZT6) phases in line with previous findings (Fig. 2, K and L) (*20*). Notably, distance traveled on active days in 48-hour cyclers was also greater when compared to Meth-naïve mice (Fig. 2M), while there was no difference between inactive days and Meth-naïve animals (Fig. 2N). We next examined the degree of straightness of the movement path in the open field arena. It has been previously shown that patients with BD with current mania would exhibit more straight-path movements over control individuals when their locomotion was traced via a room-ceiling installed video camera (*21*, *22*). Path straightness is quantified using the metric spatial *d*, which reports on the difference of spatial displacement in two-dimensional space for a given distance traveled (see Materials and Methods for details). The straighter the movement path, the lower the spatial *d*, and, consequently, spatial *d* was found to be lower in manic patients over healthy controls (*21*, *22*). We applied the spatial *d* analysis to the open field recordings of the 48-hour rhythmic mice and found that spatial *d* was lower on active days when compared to inactive days (Fig. 2J). This result is consistent with the visual inspection of the movement path, showing more circumscribed locomotion on inactive days when compared to active days (Fig. 2H). As with distance traveled (Fig. 2L), there were no differences in spatial *d* in Meth-naïve mice between their active and inactive phases (Fig. 2O), and there was no significant difference between spatial *d* measured during inactive days in 48-hour cyclers and Meth-naïve mice (Fig. 2Q). However, 48-hour cyclers on active days showed significantly lower spatial *d* as compared to Meth-naïve mice (Fig. 2P). Thus, 48-hour rhythmic mice show hyperactivity and more straight-path movement every other day, which is in line with the behavior of patients in mania, corroborating 48-hour rhythmic mice as a suitable model for mania cycling in BD.

### ^VTA^DA neurons are necessary for infradian rhythm emergence

**Fig. 3.**
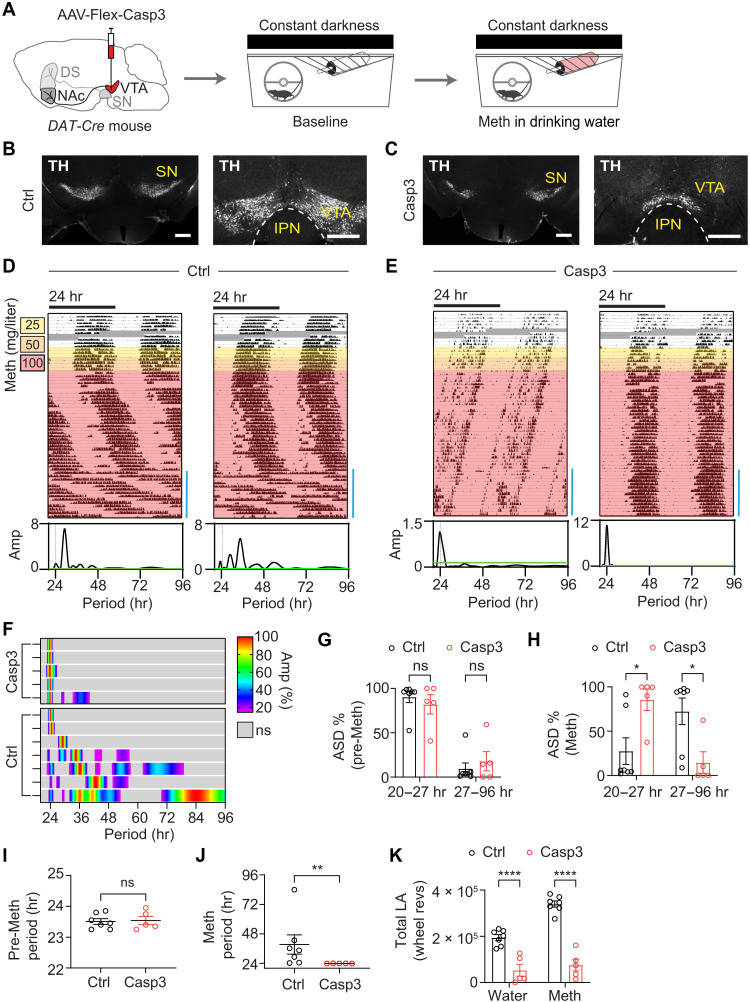
Infradian rhythm generation capacity is abrogated upon ^VTA^DA neuron ablation. (**A**) Experimental design: DA neuron ablation in the VTA via Casp3 and subsequent behavioral monitoring using running wheels. (**B** and **C**) Bilateral injection of AAV-DIO-taCasp3-TEVp into the VTA of *DAT-Cre* mice led to a loss of TH immunofluorescence in the VTA (C) compared to saline injected mice (B). IPN, interpeduncular nucleus. Scale bars, 500 μm. (**D** and **E**) Representative actograms showing running wheel activity of ^VTA^Casp3 mice and controls in constant darkness in response to escalating levels of Meth in drinking water. Lomb-Scargle periodograms below the actograms are computed for the time window indicated by the blue bar at the bottom right of the actograms. Grayed areas indicate data loss. (**F**) Composite display of periodograms from individual animals computed from the final 2 weeks of recording under Meth treatment. Periodograms are normalized to its peak amplitude. Gray shading indicates absence of significant amplitudes. (**G** and **H**) Periodogram-derived ASD of significant periodicities in the circadian (20- to 27-hour) and infradian (27- to 96-hour) period range before (G) and during the final 2 weeks of Meth exposure (H). (**I** and **J**) Periodogram-derived highest peak in the 20- to 96-hour range before (I) and during the final 2 weeks of Meth treatment (J). (**K**) Total locomotor activity (wheel revolutions) before (water) and during Meth treatment (Meth, final 2 weeks of treatment). Means ± SEM. *n* = 5 to 7. Mann-Whitney test, (I) and (J); two-way analysis of variance (ANOVA) with Bonferroni multiple comparison test, (G), (H), and (K). *P < 0.05; **P < 0.01; ****P < 0.0001.

We showed previously that chemogenetic activation of midbrain DA neurons lengthens the period of locomotor rhythms in clock-deficient mice under constant darkness conditions (*12*). Furthermore, optogenetic activation of tyrosine hydroxylase (TH)–expressing ventral tegmental area (VTA) neurons promotes wakefulness even shortly after lights on, when daily sleep pressure is highest in mice (*23*), which suggests that DA neurons of the VTA (^VTA^DA) neurons can override circadian sleep-wake control. Together, these findings are consistent with ^VTA^DA neurons acting as drivers of infradian rhythms in sleep-wake and mania-associated behaviors. To test this, we first aimed to genetically ablate ^VTA^DA neurons using a virus that Cre-dependently expresses genetically engineered Caspase 3 (Casp3) (AAV-flex-taCasp3-TEVp) (*24*) to trigger cell-autonomous apoptosis. To limit cell ablation to DA neurons, we injected the virus bilaterally into the VTA of *DAT-Cre* mice (*25*) (Fig. 3A). Subsequent immunostaining against the catecholamine cell marker TH revealed a preferential loss of TH in the VTA, but not in the adjacent substantia nigra (SN) (Fig. 3, B and C), indicative of successful ^VTA^DA neuron ablation. Notably, TH expression was found retained in some cells of the ventromedial VTA in Casp3 virus-injected (^VTA^Casp3) mice (Fig. 3C), suggestive of poor Cre activity, which we confirmed by crossing *DAT-Cre* mice with a reporter line (fig. S3, A and B) or *TH^fl/fl^* mice (fig. S3C). This recombination deficit also resonates with previous observations of lower DAT expression in TH cells of this ventromedial VTA region (*26*). Next, we monitored running wheel activity of ablated animals (^VTA^Casp3 mice) in response to Meth in constant darkness to prevent interference of endogenous rhythm generation by the light-dark cycle. A gradual increase in Meth in drinking water led to the expected emergence of an infradian component in control animals, as evidenced by the distinct pattern changes in locomotor activity (Fig. 3D) and corresponding emergence of >24-hour peaks in the periodogram (shown underneath actogram for the time span indicated by vertical blue bar). By contrast, ^VTA^Casp3 mice showed a profound loss of 2ndC induction capacity (Fig. 3E), confirmed by the lack of a rhythmicity shift into the infradian range (Fig. 3F). This was quantified by calculating the amplitude spectral density (ASD) for the circadian (20- to 27-hour) and infradian (27- to 96-hour) ranges, respectively. We chose the 27-hour mark as the bin divider because it is around the upper entrainment limit of the circadian timer (*27*) and, thus, periods above 27 hours are unlikely to result from circadian clock action. The ASD analysis confirmed a lack of a shift of rhythmic power into the infradian range in ^VTA^Casp3 mice upon Meth treatment (Fig. 3, G and H). Consistently, the highest periodogram peak shifted to infradian periods in controls, while it remained circadian in ^VTA^Casp3 animals (Fig. 3J). Notably, total activity tended to be lower in ^VTA^Casp3 mice before and during Meth treatment (Fig. 3K), suggesting an additional global effect on locomotor activity upon DA neuron ablation in the VTA, in keeping with their role in arousal maintenance (*23*). However, DA neuron ablation in the VTA did not affect circadian period based on the highest peak analysis before Meth (Fig. 3I), indicating that the central circadian pacemaker residing in the suprachiasmatic nucleus (SCN) remained functionally intact.

### ^VTA^TH is required for infradian rhythmicity

**Fig. 4.**
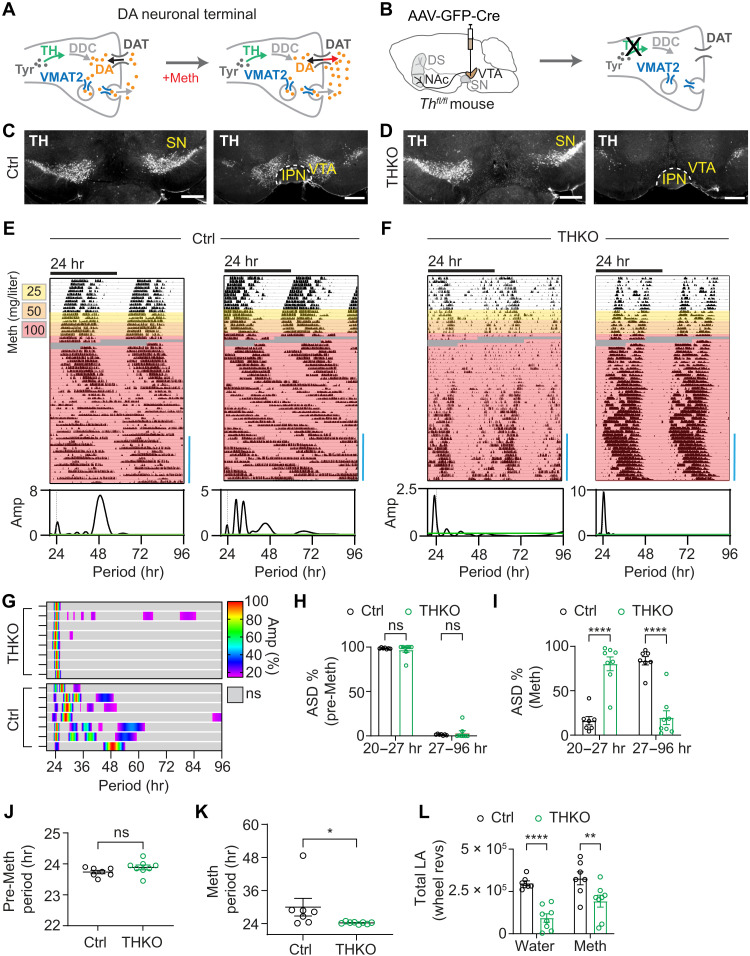
Selective disruption of *Th* in the VTA abrogates infradian rhythm generation capacity. (**A**) Schematic illustrating the Meth-mediated elevation of extracellular DA at a DA neuronal terminal. DDC, l-dopa decarboxylase. (**B**) Strategy for selective elimination of DA production in DA neurons of the VTA. (**C** and **D**) TH immunolabeling of midbrain sections from saline [(C), Ctrl] and AAV-GFP-Cre–injected animals [(D), THKO]. Scale bars, 500 μm. (**E** and **F**) Representative actograms showing running wheel activity of control and ^VTA^THKO mice in constant darkness in response to escalating levels of Meth in drinking water. Grayed areas indicate missing data. Lomb-Scargle periodograms correspond to time spans indicated by blue bar. (**G**) Composite display of normalized periodograms from individual animals corresponding to the final 2 weeks of Meth treatment. (**H** and **I**) Periodogram-derived ASD % of significant periodicities in the circadian and infradian period ranges before [(H), pre-Meth] and at the end of Meth treatment [(I), Meth]. (**J** and **K**) Periodogram-derived highest peak before [(J), pre-Meth] and at the end of the Meth treatment regimen [(K), Meth]. (**L**) Total locomotor activity before (water) and during Meth treatment (Meth). Means ± SEM. *n* = 7 to 8. Mann-Whitney test, (J) and (K); two-way ANOVA with Bonferroni multiple comparison, (H), (I), and (L). *P < 0.05; **P < 0.01; ****P < 0.0001.

Considering that Meth treatment and DAT deficiency alter DA levels (*11*) (Fig. 4A), it seemed likely that DA would be of critical importance for 2ndC induction. We therefore disrupted TH, an enzyme essential for DA biosynthesis (*28*), exclusively in ^VTA^DA neurons (Fig. 4B). Adult *Th^fl/fl^* mice (*29*) were injected with a Cre recombinase expressing adeno-associated virus (AAV) (AAV-GFP-Cre) into the VTA bilaterally, which led to a selective loss of TH in the VTA (^VTA^THKO) (Fig. 4, C and D, and fig. S4). As in the case of ^VTA^Casp3 mice, ^VTA^THKO mice also failed to produce a 2ndC in response to Meth treatment (Fig. 4, E to K), whereas the circadian period before Meth remained unaffected (Fig. 4J). Notably, ^VTA^THKO mice showed increased locomotion upon Meth treatment, indicating that these mice are not globally unresponsive to Meth (fig. S5A). Together, these results indicate that it is the DA production capacity of ^VTA^DA neurons, which is required for 2ndC emergence.

### Low locomotor activity does not prevent infradian rhythm generation

**Fig. 5.**
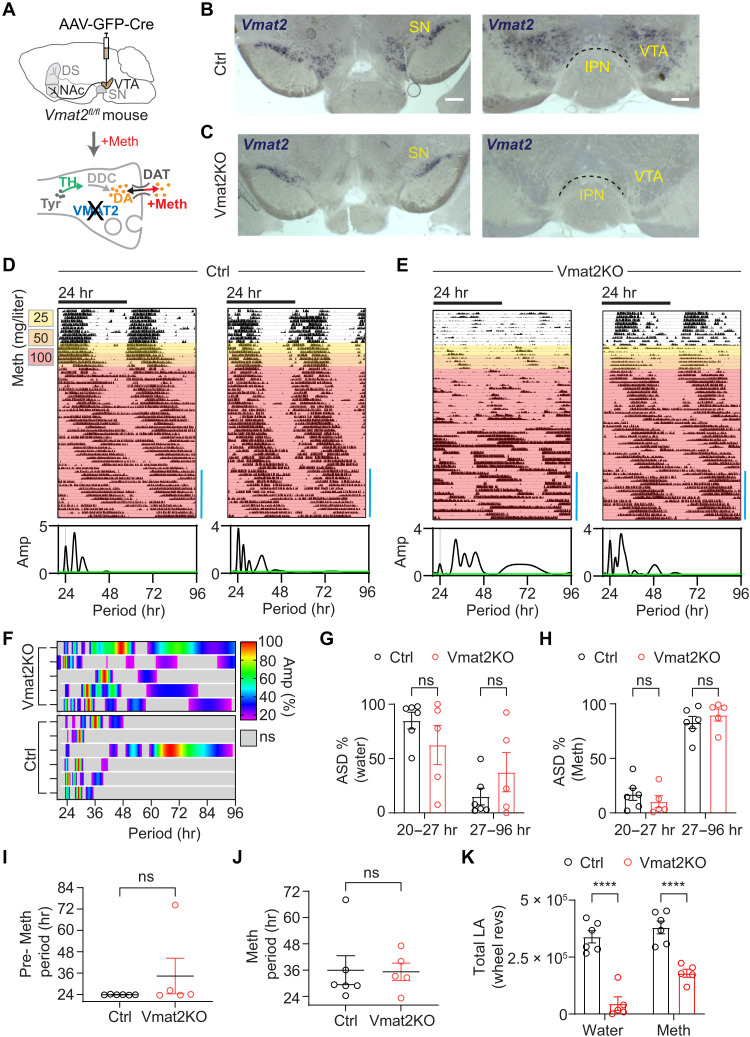
Selective disruption of *Vmat2* in the VTA does not abrogate the capacity for infradian rhythm generation. (**A**) Strategy of *Vmat2* disruption leading to loss of DA vesicular uptake and release selectively in DA neurons of the VTA. (**B** and **C**) Bilateral injection of AAV5-GFP-Cre into the VTA of *Vmat2^fl/fl^* mice leads to loss of *Vmat2* message in the VTA but not the SN. Shown are representative images of brain sections after situ hybridization with a *Vmat2* specific riboprobe of viral [(C), Vmat2KO] and saline-injected mice [(B), Ctrl)]. Blue precipitate indicates *Vmat2* message. Scale bars, 500 μm (SN) and 250 μm (VTA). (**D** and **E**) Representative actograms displaying running wheel activity of control (D) and ^VTA^Vmat2KO mice (E) in constant darkness in response to Meth in drinking water. Lomb-Scargle periodograms shown below correspond to the time span indicated by blue bar. (**F**) Composite display of normalized periodograms computed from the final 2 weeks of recording under Meth treatment. (**G** and **H**) Periodogram-derived ASD % of significant periodicities in the circadian and infradian period ranges before (G) and at the end of Meth treatment (H). (**I** and **J**) Periodogram-derived highest peak in the 20- to 96-hour range before [(I), pre-Meth] and at the end of Meth treatment [(J), Meth]. (**K**) Total locomotor activity before (water) and during Meth treatment (Meth). Means ± SEM. *n* = 5 to 6. Mann-Whitney test, (I) and (J); two-way ANOVA with Bonferroni multiple comparison, (G), (H), and (K). ****P < 0.0001.

Consistent with ^VTA^Casp3 mice, ^VTA^THKO mice also showed a reduction in overall locomotor activity when compared to controls (Fig. 4L), which could be viewed as a contributing factor to the failed 2ndC emergence in response to Meth. To address this possible confounder, we created mice deficient of the vesicular monoamine transporter 2 (Vmat2) specifically in ^VTA^DA neurons, which consequentially abrogates DA vesicular uptake and, thus, release (Fig. 5A).

Bilateral injections of AAV-GFP-Cre into the VTA of *Vmat2^fl/fl^* mice led to a preferential loss of *Vmat2* transcripts in the VTA (^VTA^VMAT2KO) (Fig. 5, B and C), while expression in the SN, raphe nucleus, and locus coeruleus remained intact (Fig. 5C and fig. S6, A and B). As was the case for ^VTA^Casp3 and ^VTA^THKO mice, ^VTA^Vmat2KO mice also exhibited a marked reduction in spontaneous locomotor activity (Fig. 5K), while the circadian period before Meth remained unaffected again (Fig. 5I). However, contrary to the other models, ^VTA^Vmat2KO mice retained the ability to produce 2ndCs (Fig. 5, D and E). Both the highest peak (Fig. 5, I and J) and spectral energy distribution (Fig. 5, F to H) showed a shift toward infradian periods for both controls and ^VTA^Vmat2KO mice. Notably, as in the case of ^VTA^THKO mice, ^VTA^Vmat2KO animals also showed increased total locomotor activity in response to Meth (fig. S5, A and B); however, in contrast to the former, Vmat2KO mice are able to produce 2ndCs. Similar results were obtained with *Vmat2^fl/fl^* mice that were crossed to *DAT-iCreER* mice (*30*). Tamoxifen treatment of *DAT-iCreER × Vmat2^fl/fl^* mice led to a selective loss of *Vmat2* in DA neurons across the midbrain and a profound loss of locomotor activity (fig. S6, C to F). However, subsequent exposure to Meth not only increased activity in form of distinct locomotor bouts but also led to the emergence of 2ndCs (fig. S6F). For further confirmation of the Vmat2KO findings, we injected the VTA of *DAT-Cre* mice with an AAV expressing tetanus toxin light chain (TetTox) (*31*) in a Cre-dependent manner (AAV-DIO-GFP-2A-TetTox) to synaptically silence DA neurons (*31*). TH-positive fibers of the nucleus accumbens (NAc) shell were found to comprehensively costain for the viral green fluorescent protein (GFP) reporter indicating successful targeting of NAc-projecting ^VTA^DA neurons by the virus (fig. S7, A and B). As with ^VTA^Vmat2KO mice, we found ^VTA^TetTox mice to exhibit reduced locomotor activity at baseline (fig. S7, C and D), while their 2ndC induction capacity was fully retained (fig. S7, C to E). Together, these findings demonstrate that a general, sustained reduction in spontaneous locomotion does not preclude generation of a 2ndC in response to Meth and Meth-induced hyperlocomotion is not a predictor of 2ndC generation capacity.

### Period lengthening by chemogenetic activation of ^VTA^DA neurons is counteracted by antipsychotic treatment

**Fig. 6.**
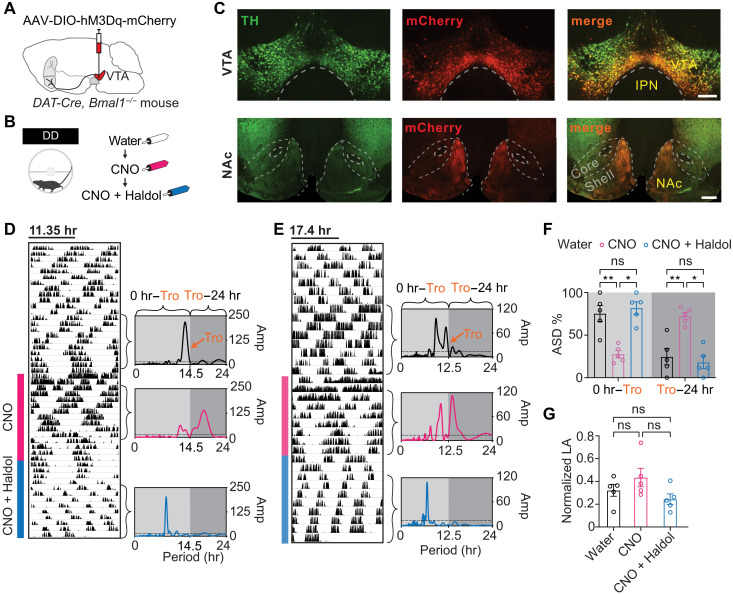
Period shortening of ^VTA->NAc^DA neuron–driven locomotor rhythms by antipsychotic treatment. (**A** and **B**) Experimental regimen of locomotor rhythm monitoring in constant darkness and CNO treatment upon viral delivery of a chemogenetic actuator into DA neurons of the VTA. (**C**) Immunofluorescence images of VTA and NAc sections labeled for mCherry (red) and TH (green) from *DAT-Cre × Bmal1^−/−^* mice injected with AAV-DIO-hM3Dq-mCherry in the VTA. Core and shell indicate respective subregions of the NAc. Scale bars, 500 (NAc) and 250 μm (VTA). (**D** and **E**) Representative, modulo-plotted actograms showing locomotor responses to CNO in drinking water, followed by CNO + Haldol. Periodograms are computed from indicated time windows. “*Tro*” indicates the trough after the dominant periodogram peak during water treatment. The 0-hour to Tro periodogram segment therefore captures the periodicities that dominate at baseline (water-only treatment). Dashed line indicates significant threshold for rhythmicity. (**F**) Percentage of total ASD allocated to 0 hours to *Tro* and *Tro* to 24 hours, respectively. (**G**) Cumulative locomotor activity over 96 hours normalized to the sum of the three 96-hour time windows indicated by brackets. Means ± SEM. *n* = 5. Two-way ANOVA with Bonferroni multiple comparison. *P < 0.05; **P < 0.01.

To assess whether activation of NAc projecting ^VTA^DA neurons is sufficient for DO action, we injected *DAT-Cre × Bmal1^−/−^* mice with the Cre-activatable AAV-DIO-hM3Dq-mCherry (*32*) into the ventral VTA to drive expression of the chemogenetic activator hM3Dq specifically in TH neurons (Fig. 6, A and B). The clock-deficient *Bmal1^−/−^* background was chosen to observe unobstructed operation of the DO in the absence of a light-dark cycle and circadian timer that may obscure any DO action triggered by a chemogenetic actuator. This is under the assumption that chemogenetic activation is less powerful than Meth to drive DO rhythmicities. The mCherry expression pattern confirmed the expected preferential transduction of TH neurons in the ventral VTA and corresponding processes in the NAc medial shell (Fig. 6C). Addition of the hM3Dq-ligand clozapine *N*-oxide (CNO) to the drinking water lengthened locomotor period (Fig. 6, D to F). Next we applied haloperidol (Haldol), a common antipsychotic and dopamine receptor D2 inverse agonist (*33*), which we have previously shown to counteract not only Meth-mediated period lengthening in clock-deficient mice but also 2ndC emergence in clock-intact mice (*12*). Haldol supplementation reversed the CNO-mediated effect on locomotor period in the hM3Dq-expressing mice (Fig. 6, D to F), suggesting that CNO acts on the same oscillatory process as Meth treatment or DAT disruption. Notably, neither CNO nor CNO + Haldol treatment significantly altered total locomotor activity (Fig. 6G), further suggesting that the change in period is not a mere consequence of a change in overall activity.

### Infradian rhythm emergence requires NAc-projecting DA neurons

**Fig. 7.**
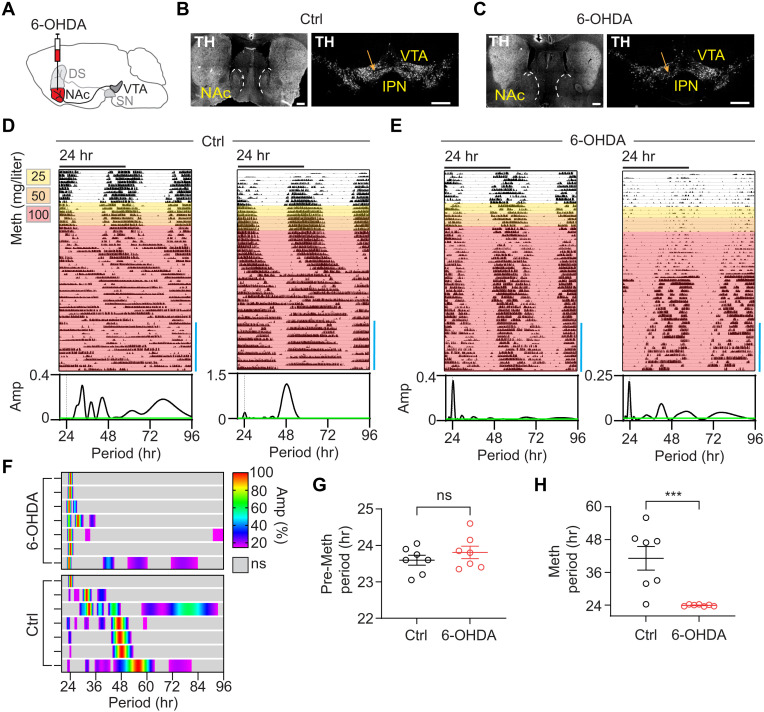
Loss of infradian rhythm generation capacity upon NAc-projecting DA neuronal ablation and two-oscillator model for BD cycling. (**A**) 6-OHDA was bilaterally injected into the NAc to ablate dopaminergic processes. (**B** and **C**) TH immunolabeling of striatal and midbrain sections from saline-injected (B) and 6-OHDA–injected (C) animals. Scale bars, 500 μm. (**D** and **E**) Representative actograms showing running wheel activity of saline-injected (D) and 6-OHDA–injected (E) mice in constant darkness in response to escalating doses of Meth in drinking water. Lomb-Scargle periodograms correspond to the time interval indicated (blue bars). (**F**) Composite display of normalized periodograms from individual animals computed from the final 2 weeks of recording under Meth treatment. (**G** and **H**) Periodogram-derived highest peak in the 20- to 96-hour range before [(G), pre-Meth] and at the end of Meth treatment [(H), Meth]. Means ± SEM. *n* = 7. Mann-Whitney test. ***P < 0.001.

The chemogenetic findings support a role of NAc-projecting DA (^NAc^DA) neurons in noncircadian rhythm generation. We therefore wondered whether these neurons are necessary for infradian rhythm emergence and, thus, injected the catecholaminergic neurotoxin 6-hydroxydopamine (6-OHDA) bilaterally into the NAc medial shell region to selectively ablate DA neurons that project to the NAc (^NAc^6-OHDA) (Fig. 7A). Immunohistochemical examination of ^NAc^6-OHDA mice showed the expected preferential loss of TH signal in the NAc and corresponding reduction but not complete elimination of the TH immunosignal in the VTA (Fig. 7, B and C), as expected, given that the NAc is only one of several ^VTA^DA neuronal targets (*34*). Mirroring the behavior of ^VTA^Casp3 and ^VTA^THKO mice, ^NAc^6-OHDA animals largely failed to produce a 2ndC in response to Meth (Fig. 7, D to H, and fig. S8, A and B). In line with the other models, the circadian period before Meth remained unaffected (Fig. 7G), while locomotor activity tended to be lower under pre-Meth conditions (fig. S8C).

## DISCUSSION

We previously showed that global elimination of DAT can lead to the emergence of a 2ndC (*12*), i.e., a manipulation that selectively yet broadly targets DA neurons is sufficient for infradian rhythms induction. Our new data now demonstrate the necessity of NAc-projecting DA neurons of the VTA for infradian rhythm emergence arguing for a role as infradian rhythm generators.

Both Meth treatment and DAT knockout (DATKO) result in 2ndC emergence and elevation in extracellular DA, yet the latter seems to be achieved by different mechanism: a reverse-acting DAT in the case of Meth and vesicular DA release without reuptake in the case of *DAT^−/−^* mice. The preservation of Meth-induced infradian rhythmicity in the absence of DA-vesicular release (*Vmat2KO*) or general synaptic silencing (TetTox experiment) further underscores that while DA release by ^VTA^DA neurons is of critical importance for 2ndC induction, the actual release mode is not. The TetTox-silencing experiment also indicates that vesicular neurotransmitter release in general by ^NAc^DA terminals is dispensable for infradian rhythm generation. Notably, reverse action of the DAT in response to amphetamines has been already demonstrated in global *Vmat2^−/−^* mice, where amphetamine treatment extends survival of the knockout mice, which, otherwise, die shortly after birth (*35*).

Our finding that the period of 2ndC reaches far into infradian range challenges the currently held view that the oscillator that Meth engages is circadian in nature, as reflected in the wide-spread use of the term “MASCO” (Meth-sensitive circadian oscillator) as descriptor for this process (*7*, *13*). Considering our previous findings that 2ndC emergence does not only require Meth but, instead, can also result from DAT disruption (*12*) along with our data presented here (Fig. 1), we now propose to use dopamine oscillator or DO to identify the oscillator that drives infradian rest and arousal cycles and relies on DA production. This more general descriptor should also take precedence over the use of the term “dopaminergic ultradian oscillator” we previously coined for this oscillator (*12*), given the wide range of periods that it can adopt.

Forty-eight–hour rhythms in rest and arousal could be considered a peculiar finding due to the scarcity of its accounts in the animal and human literature (*13*, *36*). However, a recent BD case study showed that pausing treatment of the widely used antipsychotic aripiprazole resulted in the emergence of 48-hour sleep-wake cycles with sleep cycles again normalizing to daily (24-hour) rhythms after drug reinstatement (*37*). This demonstrates that standard medication can obscure cycling in BD, which, in turn, suggests that periodic state switches including 48-hour cycling may be much more widespread in BD than currently acknowledged. Such a “blotting-out” effect by drug therapy in the context of cycling behavior was already suggested nearly a century ago (*3*).

In further support of 48-hour rhythmic mice representing a valid model for cycling in BD, we found them to display hyperactivity and more straight-path movements on days with short sleep. While these data indicate face validity for cyclical BD mania, there is also evidence for predictive validity as antipsychotic treatment corrects infradian rhythmicity in both Meth and DATKO models much in line with the abovementioned case report (*12*). Last, high-dose Meth has been recently reported to trigger bipolar mood cycling de novo (*38*), which can be viewed as evidence for construct validity. Notably, increased activity/energy has been elevated to gate criteria for the diagnosis of BD with the introduction of the Diagnostic and Statistical Manual of Mental Disorders, Fifth edition (DSM-5) (*19*), which was previously limited to elevated or irritable mood. While there are other behavioral tests used to evaluate animal models of BD for the (hypo)manic state (*39*–*41*), these tests by and large do not address mood. Moreover, there seems to be stronger evidence for increased activity/energy as more consistent symptoms of (hypo)mania and may even be the most defining feature of the disorder (*42*–*45*), adding specific strength to the behavioral parameters we measured to validate our mouse model.

These accounts together with the fact that the dopamine system is one of the principal BD treatment targets (*46*) argue that the 48-hour rhythmic mice characterized here represent a bona fide model for 48-hour cycling in BD. This, in turn, would then suggest that NAc-projecting DA neurons serve as a substrate not only for murine infradian rhythm production but also for 48-hour cycling in BD.

While there exists a considerable number of mouse models for mania or depression (*39*–*41*), animal models for cycling or mood-state switching are scarce (*47*). However, understanding state switching mechanistically is considered the “holy grail” of BD (*48*). Forty-eight–hour cycling mice may therefore critically help elucidate the cellular and molecular basis of this disorder.

**Fig. 8.**
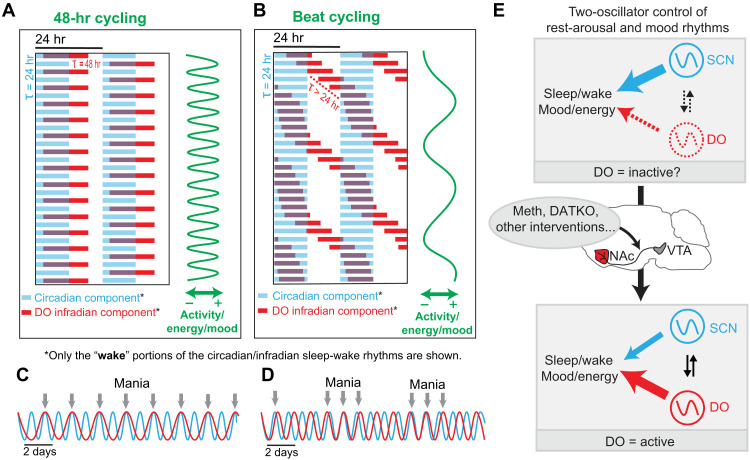
Two-oscillator model for BD cycling. (**A**) Model actogram of daily wake periods alongside fluctuations in activity/energy/mood at a DO period of 48 hours. (**B**) Daily wake periods reflective of a DO operating at a frequency nonharmonious to the SCN clock. (**C** and **D**) Phase-locking or in-and-out of phase “beating” of the DO and SCN clock when frequencies are harmonious (C) or nonharmonious (D) resulting in mania-associated behavioral cycling. (**E**) Upon “activation,” the DO influences/dominates sleep-wake and mood/mania-associated behavioral rhythms.

Mood switching in BD with great regularity has been also reported at periodicities much longer than 48 hours, such as weeks or even months (*49*–*51*), and it has been already proposed that this BD rapid cycling could be due to the periodic in- and out-of-phase “beating” of two rhythmic processes that deviate in their frequencies (*52*). Given what we know about the DO, it seems very plausible that this oscillator and the circadian clock represent those rhythmic processes with the beat frequency and, thus, mood switch frequency determined by the period of the DO, which is uniquely tunable. Meth-treated mice that show an infradian component with periods shorter than 48 hours provide evidence for beat cycling with regard to daily locomotor activity (fig. S9). A similar pattern of locomotor activity changes is also found in rapid cycling BD [see figure 1 in (*51*)], with daily locomotor activity varying in synchrony with mood, in support of the proposed beating mechanism as the basis for cycling in BD (Fig. 8).

Our findings specifically support a role of NAc-projecting DA neurons in the cycling aspect of BD. In this context, a recent positron emission tomography imaging study using a DAT ligand as a tracer revealed lower DAT availability in manic patients versus controls, selectively in striatal regions, including the NAc (*53*). Given that the DAT is exclusively expressed by DA projections in the NAc, these data provide further credence for the ^NAc^DA projections as site or drivers of cyclicity in BD. In line with this recent positron emission tomography data, diffusion tensor imaging of a rapid cycling female patient who exhibited 2 weeks of mania in association with each menstrual cycle, showed increased fractional anisotropy in the manic state, which was largely restricted to the NAc region when compared to the patient’s preceding and succeeding euthymic states or to unaffected control individuals (*54*). This latter study can be viewed as first evidence for a correlate of BD cycling in human brain activity that intriguingly centers on the NAc, in further support of NAc-projecting DA neurons as drivers of infradian rhythms and bipolar cycling.

While we provide evidence that the DO can produce infradian rhythms at a wide range of periods upon manipulation of the DA system, there is the question about the DO’s state in intact laboratory rodents without a challenge und in healthy humans. While the DO may adopt noninfradian periodicities under these conditions, it could as well be inoperative with temporal sleep-wake regulation solely resting on the circadian timer. In this scenario, the DO would constitute an “activatable” rhythm generator that can jump into action when challenged by, e.g., psychostimulants or in patients with BD (Fig. 8E). Because of its robust rhythm generation capacity, it seems very likely that the DO has functions beyond the pathological context—whether as a constitutive active or activatable entity—which, however, have yet to be elucidated.

There are limitations to this study. We show infradian rhythmicity in behavioral output but not on the neurophysiological level, which could further strengthen our findings. We used saline-injected mice as controls for virally mediated DA cell ablation and TH disruption. Therefore, we cannot rule out that the viruses may have contributed to the observed loss of infradian capacity beyond their payload action. However, in the case of the AAV-GFP-Cre virus, which was used to disrupt TH, we could show that it does not affect infradian rhythm induction when used to disrupt *Vmat2* across the VTA. We demonstrate that chemogenetic activation of ^VTA^DA neurons including those projecting to the NAc in clock-deficient (*Bmal1^−/−^*) mice induces locomotor period lengthening, which is counteracted by Haldol. We chose this mouse model considering that DO activation by this modality is weaker compared to DAT disruption or Meth treatment and, thus, may not lead to a discernible 2ndC in the presence of the circadian clock. The observed period lengthening was induced by treatment with CNO, which engages the chemogenetic actuator hM3D. While it is possible that CNO can affect behavior independent of this actuator, it seems unlikely that it can account for the observed period effect in rest and arousal, as CNO has been previously shown to not affect overall proportions of daily wake and sleep in mice even at doses 10-fold higher than used here (*55*). Our data argue that infradian rhythmicity relies on NAc-projecting DA neurons. Given that the NAc is divided into functionally distinct subterritories (core, medial, and lateral shell), it is conceivable that the DA neurons that act as infradian drivers are targeting only one of these NAc subregions.

## MATERIALS AND METHODS

### Study design

The overall aim of this study was to characterize the neural substrate of the oscillator process that drives infradian rhythms in rest:activity in mice and link it to mood-associated behaviors and sleep cycling in BD. To this end, we used mice that received Meth via drinking water to induce infradian rhythmicity. Groups of genetically modified mice received intracranial AAV/neurotoxin injections to manipulate or label midbrain DA neurons and were then assayed for their ability of rhythm lengthening using running wheel or general locomotor activity as read outs. Infradian rhythmic mice were also subjected to behavioral tests.

Sample sizes for all experiments are indicated in the figure legends and are similar to those used by other researchers in the field. Age-matched mice of both sexes were used and randomized to each experimental group. None of the animals were excluded from the analysis unless they died before brain collection for histological confirmation. All animal procedures were carried out in accordance with the recommendations of the Canadian Council on Animal Care and have been approved by the local McGill University Animal Care Committee (DOUG-5945).

### Animals

*Bmal1^−/−^* mice (*56*) were crossed to *DAT-Cre* mice (*25*) to enable viral targeting of DA neurons. *Th^flox/flox^* mice (*29*) were used for viral-mediated *Th* disruption in the VTA. *Slc18a2^flox/flox^* mice (*30*) were virally targeted for midbrain *Vmat2* disruption or crossed to *DAT-iCreER* (the Jackson Laboratory, stock #016583) mice for body-wide, DA neuron–specific *Vmat2* disruption during adulthood. Mouse lines were on a C57BL/6 background.

### Locomotor activity monitoring and sleep analysis

Animals were individually housed in running-wheel cages in light-tight cabinets in constant darkness. Locomotor activity was recorded continuously using ClockLab data collection software (ClockLab, Actimetrics). ClockLab analysis software was used to generate actograms displaying binned running-wheel revolutions per 5 min (0.12 hours), Lomb-Scargle (*57*) periodograms, and heatmap displays of Morlet CWTs and associated wavelet ridge plots.

For passive infrared recordings, animals were individually housed in standard cages. Locomotion was recorded at 0.25 Hz and binned at 1-min intervals using custom software. Locomotion data were transformed into actogram displays using MATLAB (The MathWorks) custom scripts (https://github.com/clementbourguignon/Activity-monitoring). Sleep counts were generated by assigning a score of 1 to any 1-min bin with a value of 0, in concordance with (*17*, *18*). For sleep analysis during 48-hour cycling, we selected 4-day time spans of 48-hour rhythmicity (fig. S2) for each animal using spectral power quantification of circadian (22- to 26-hour) versus infradian (44- to 52-hour) range frequencies as guide for selection.

### In situ hybridization

In situ hybridization was performed as described previously (*58*). Briefly, fixed brains collected after intracardiac perfusion (Z-fix, Anatech Ltd.; 10% aqueous buffered zinc formalin) were cut at 25 μm using a cryostat (Leica) and stored at −80°C until hybridization. Sections were hybridized overnight at 60°C to a digoxigenin-labeled riboprobe targeting the coding regions of mouse *Vmat2 (*nucleotides 141 to 255 of the *Slc18a2* mRNA; GenBank, NM_172523.3).

### Immunohistochemistry

Immunostaining was performed as previously described (*59*). Briefly, mice were deeply anesthetized and transcardially perfused with 10% formalin (Z-fix, Anatech Ltd.). Brains were postfixed in 10% formalin overnight and then incubated in 30% sucrose in saline for at least 24 hours. Brains were cut at 40 μm using either a cryostat (Leica) or vibratome (Leica, VT 1200S) and collected in three or four series per brain, respectively. For immunohistochemistry, sections were rinsed in phosphate-buffered saline (pH 7.4), incubated in blocking solution (1% goat or donkey serum in 0.3% Triton X-100 in phosphate-buffered saline) for 1 hour, followed by incubation with the primary antibody in blocking solution at 4°C overnight. Sections were then incubated in secondary antibodies for 2 hours at room temperature. Sections were mounted on superfrost slides (VWR), coverslipped with VECTASHIELD mounting medium with 4′,6-diamidino-2-phenylindole (VectorLabs) and imaged by fluorescence microscopy. Primary and secondary antibodies were used at the following dilutions: rabbit anti–red fluorescent protein (1:1000; Rockland, catalog no. 600-401-379, RRID: AB_2209751) to enhance detection of mCherry expression and mouse anti-TH (1:1000; Millipore, catalog no. MAB318, RRID: AB_2201528) for TH detection; Alexa Fluor 488 and Alexa Fluor 568 conjugates (Thermo Fisher Scientific) were used as secondary antibodies at a dilution of 1:500.

### Pharmacology

#### Tamoxifen

To activate Cre recombinase, *DAT-iCreER × Vmat2^fl/fl^* mice were intraperitoneally (ip) injected with tamoxifen [Sigma-Aldrich; dissolved in a 1:9 (v/v) ethanol:sunflower oil mix to a final concentration of 30 mg/ml] twice daily for five consecutive days. The first dose (0.6 mg) was given 3 hours after lights on and the second (0.75 mg) 1 hour before lights off.

#### Meth, Haldol, and CNO

Stock solutions of (+)-Meth hydrochloride (25 to 100 mg/liter; National Institute of Mental Health, Bethesda, MD), Haldol (11.25 mg/ml; Sigma-Aldrich), and CNO (15 mg/liter; National Institute of Mental Health, Bethesda, MD) were prepared using drinking tap water. Haldol was dissolved by stirring at 40°C. Drugs were added to the drinking water to reach concentrations as indicated.

### ASD and CWT computations

To determine the prevalence of oscillations in a given period range, we calculated the sum of amplitude of all significantly rhythmic periodicities (α = 0.001) in the Lomb-Scargle periodogram, that is, the power spectral density. Densities for circadian (20- to27-hour) or infradian (27- to 96-hour) ranges were then normalized by division with the total significant spectral density (20- to 96-hour) and expressed as percentage according to the following formula

$$\%\text{ASD}\;\left(\text{circadian}\right)=\frac{\sum_{20h}^{27h}P\left(f\right)\;\text{for}\;N\;\text{days}\;}{\sum_{20h}^{96h}P\left(f\right)\;\text{for}\;N\;\text{days}\;}\;\times \;100$$

$$\%\text{ASD}\;\left(\text{infradian}\right)=\frac{\sum_{27h}^{96h}P\left(f\right)\;\text{for}\;N\;\text{days}\;}{\sum_{20h}^{96h}P\left(f\right)\;\text{for}\;N\;\text{days}\;}\;\times \;100$$

where *P*(*f*) represents the power calculated by the Lomb-Scargle method (*57*).

CWTs and scale-averaged wavelet spectra were computed using the MATLAB Wavelet Toolbox with Morse wavelets and 24 voices per octave. The windows used for computing the scale averaged wavelet spectrum were 20 to 27 and 27 to 72 hours, respectively.

To quantify the effect of chemogenetic activation in AAV-DIO-hM3Dq-mCherry (*32*)–injected *DAT-Cre × Bmal1^−/−^* mice on locomotor period, changes in power distribution were calculated as follows: First, we identified the dominant peak in the periodogram computed from the 96-hour time span before CNO treatment. We then used the trough that followed the dominant peak as reference point for quantifying shifts in power distribution (indicated by Tro in Fig. 6). Accordingly, we calculated the percentage of total ASD allocated to the periodogram segments “0 hours to Tro” and “Tro to 24 hours” of this trough, respectively. The “Tro” can be considered analogous to the 27-hour mark dividing the periodogram in circadian (20- to 27-hour) versus infradian (27- to 96-hour) ranges in the context of Meth exposure in circadian intact mice. Note that in contrast to the circadian period in intact animals, the ultradian locomotor period profoundly varies among *Bmal1^−/−^* animals, typically in the 2- to 8-hour range. Therefore, a fixed reference point such as 27 hours in the case of circadian intact mice cannot be used and, hence, the use of Tro whose position is dictated by the ultradian period/spectral power, which is specific to each *Bmal1^−/−^* mouse.

### Virus and 6-OHDA injections

Mice were anaesthetized with isoflurane and placed in a stereotaxic apparatus (David Kopf Instruments). Recombinant AAV vectors were bilaterally injected into to the VTA area (coordinates from bregma: anterior-posterior, −3.44 mm; dorsal-ventral, −4.40 mm; lateral, ±0.48 mm; through a cannula (33 gauge; Plastics One) at a flow rate of 0.1 μl/min for 3 min (0.25 to 0.3 μl of total volume per side) using a syringe pump (Harvard Apparatus). Mice were subsequently maintained in individual housing for at least 2 weeks before CNO treatment and/or locomotor activity recording. Viruses were as follows: AAV5-hSyn-GFP-Cre (University of North Carolina at Chapel Hill Vector Core; titer, 3.5 × 10^12^ genome copies (gc)/ml; diluted 1:1 with saline), AAV5-flex-taCasp3-TEVp (*24*) (University of North Carolina at Chapel Hill Vector Core; titer, 4.6 × 10^12^ gc/ml), AAV8-hSyn-DIO-hM3D(Gq)-mCherry (*32*) (Molecular Tools Platform, CERVO Brain Research Center, Laval; titer, 7.9 × 10^12^ gc/ml), and AAV8-hSYN1-DIO-GFP-2A-TetTox (University of Michigan Vector Core; titer, 8.22 × 10^13^ gc/ml). The AAV-hSYN1-DIO-GFP-2A-TetTox plasmid was generated by polymerase chain reaction amplifying the GFP-2A-TetTox cassette (from Addgene plasmid #166603, gifted by M. Myers Jr.) and introducing Asc I and Bsi WI enzyme sites to the 5′ and 3′ ends, respectively. Following enzyme digestion, the GFP-2A-TetTox insert was ligated into an AAV-hSYN1-DIO backbone (from Addgene plasmid # 44361, gifted by B. Roth), which had been digested with Asc I and Bsr GI.

6-OHDA (concentration, 10 μg/μl) was injected in the medial NAc region (coordinates from bregma: anterior-posterior, +1.10 mm; dorsal-ventral, −4.40 mm; lateral: ±0.50 mm) at a flow rate of 0.1 μl/min for 2.5 min (2.5 μg per side); equal volume of saline was injected in the control group. Each animal received desipramine hydrochloride (25 mg/kg, ip), 30 min before the surgery to spare norepinephrine neurons from 6-OHDA–mediated neurotoxicity.

### OFT and video analysis

The OFT was carried out in a transparent box (50-cm by 50-cm floor space) over 10 min per animal at ZT0 to ZT6 or ZT12 to ZT18 in mice with no Meth and at circadian time 0 (CT0) to CT6 in Meth-exposed mice exhibiting 48-hour cycling. All experiments were carried out under constant dim light conditions, and Camera Module HBVCAM-F20216HD (Walfront Store) was used to video record the behavior at 30 frames/s. The location tracking module of ezTrack (*60*, *61*) was used to extract the animal position (*x*-*y* coordinates) and total distance traveled.

### Spatial *d* computation

Spatial *d* was calculated on the basis of fractal analysis as described before (*62*). Briefly, the animal’s spatial trace (derived from the frame-by-frame spatial displacement) extracted from the 10-min OFT recording was divided into 2-cm segments, and only the first frame of each of the 2-cm segment associated video frames was retained. We considered these frames as “microevents” in accordance to Paulus and Geyer (*62*) and used them for further analysis. We then measured the animal’s spatial displacement at different resolutions *k* (1, 2, 4, and 8) to compute the total length (*L_k_*) of the trace at each resolution. Thus, *L_k_* is the sum of the distances between the first and *k* + first microevent, followed by distance between *k* + first and 2*k* + first microevent and so forth. Next, log_2_(*L_k_*) versus log_2_(*k*) was plotted, and a nonlinear curve fit using least-squares regression was applied (Prism, GraphPad), with spatial *d* representing the (−) slope of this fit.

### Statistical analysis

Data are presented as means ± SEM. One-way and repeated-measures, two-way analysis of variance (ANOVA) followed by a Bonferroni post hoc test, Student’s *t* test, Mann-Whitney *U* test, and Wilcoxon’s test were used as indicated in figure legends.
