# Supplementary material for: Mesolimbic dopamine neurons drive infradian rhythms in sleep-wake and heightened activity state
Source: Sci Adv. 2025 Jan 1;11(1):eado9965. doi: 10.1126/sciadv.ado9965 (PMC11694791; doi:10.1126/sciadv.ado9965)
Supplement: Supplementary file 1 — Figs. S1 to S9 [file sciadv.ado9965_sm.pdf]

Science Advances
AAAS

Supplementary Materials for

Mesolimbic dopamine neurons drive infradian rhythms in sleep-wake and heightened activity state

Pratap S. Markam *et al.*

Corresponding author: Kai-Florian Storch, florian.storch@mcgill.ca

Sci. Adv. **11**, eado9965 (2025)
DOI: 10.1126/sciadv.ado9965

This PDF file includes:

Figs. S1 to S9

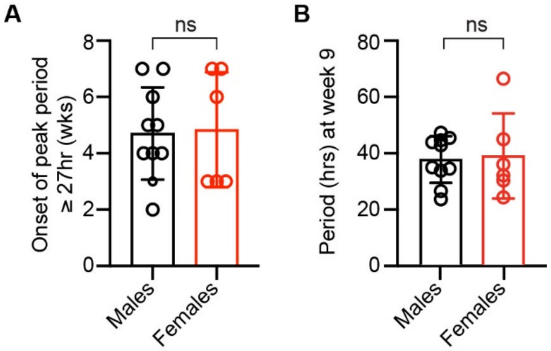

fig. S1. Infradian locomotor rhythm emergence in male and female mice. (A) Time to infradian rhythm onset in weeks post Meth exposure. Shown is the time of emergence of rhythms with a period ≥27hrs post switch to Meth containing drinking water. (**B**) Periodogram peak period at 9 wks post Meth exposure onset. Note that some animals show circadian range periods at the 9 wk mark due to infradian rhythm instability. Mice were housed in DD, locomotor activity rhythms were assessed using running wheels. Mean ±SEM. n= 6-10. Mann-Whitney U Test; ns, not significant.

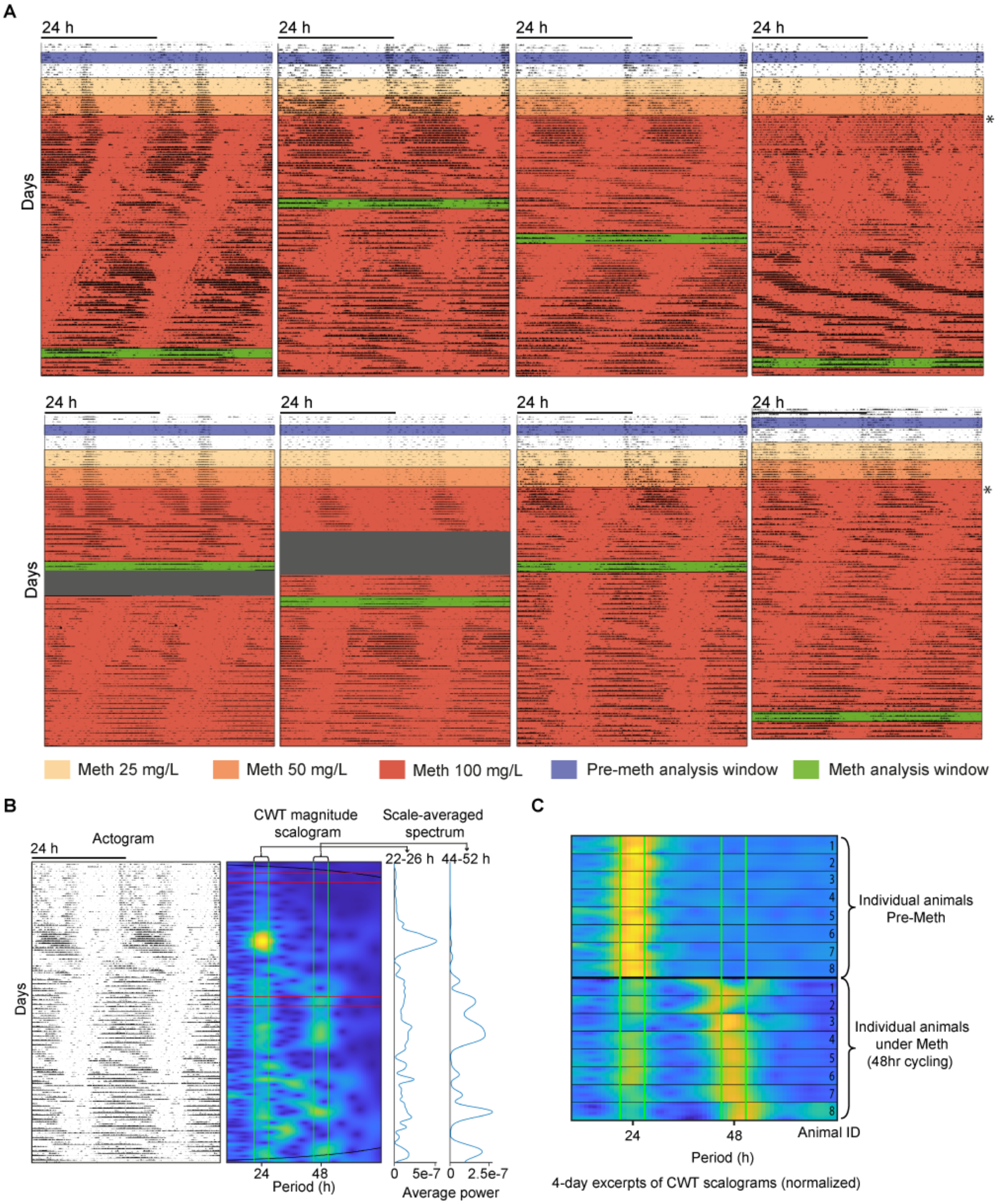

fig. S2. Meth-induced 48-hour rhythmicity in general cage activity. (**A**) Actograms displaying locomotor activity of a cohort of single-housed animals recorded by PIR sensors. Note that every animal

of the cohort tested (N=8) was able to produce a 48hr rhythmic component in response to Meth supplementation of the drinking water. Asterisk (right next to actograms) marks transition into constant darkness. (**B**) Selection of time spans for sleep analysis during 48hr cycling. Representative actogram showing locomotor activity from PIR data, along-side the CWT scalogram and scale-averaged spectral power for the circadian (22-26hr) and infradian (44-52hr) period ranges (also demarcated by green lines in the scalogram). Red lines demarcate the 4d-time span used at baseline (pre-Meth) and during Meth treatment. Selection of the second time span (Meth) was guided by the presence of a meaningfully strong peak in the 44-52hr spectral power trace. (**C**) Group display of selected time spans used for analysis.

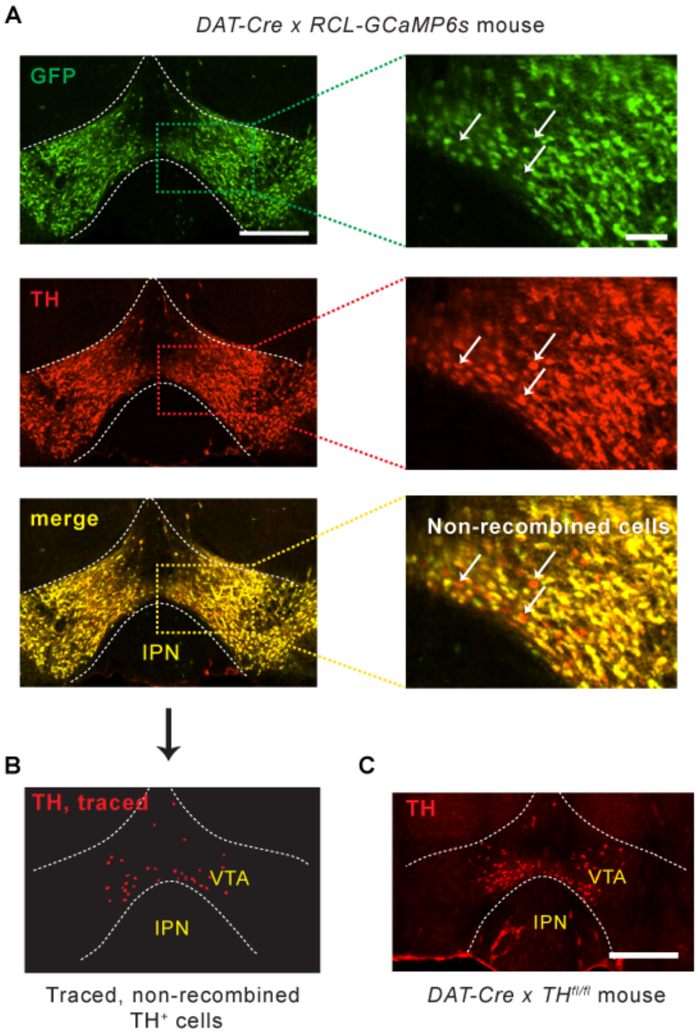

fig. S3. Assessment of Cre activity in the VTA of the *DAT-Cre* mouse line. (**A**) Some of the TH+ cells that are preferentially located in the ventral VTA apical to the IPN lacked GCaMP6s expression in *DAT-Cre x RCL-GCaMP6s* mice. Shown are immunofluorescence images of the VTA stained with antibodies against TH and GFP, respectively. Images on the right show enlargements of the boxed areas of the images on the left. (**B**) Selective display of non-recombined (GFP-negative) cells that were manually identified in (A). (**C**) TH immunostaining in the VTA of a *DAT-Cre x* $TH^{fl/fl}$ mouse. IPN, interpeduncular nucleus. Scale bar, Scale bars, 500 µm (A, left and C) and 100 µm (A, right).

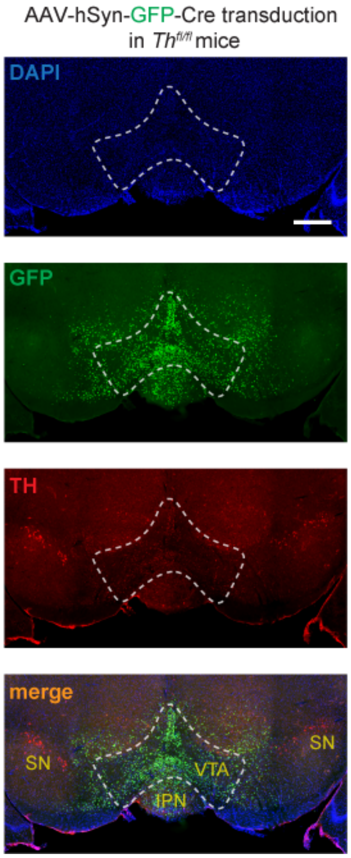

fig. S4. AAV-GFP-Cre viral spread is limited to the VTA region. Midbrain GFP/TH immunofluorescence after bilateral injection of AAV-GFP-Cre into the VTA of a $Th^{fl/fl}$ mouse. The nuclear GFP signal spread indicates that viral transduction was largely limited to VTA, sparing the SN. Scale bar, 500 μm.

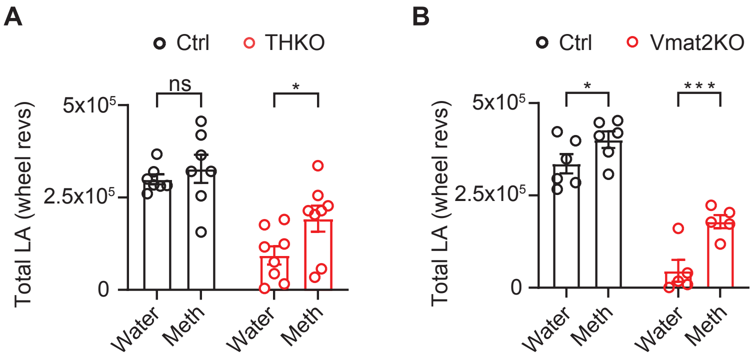

fig. S5. Effect of Meth on total locomotor activity in ^{VTA}THKO and ^{VTA}Vmat2KO mice. Total locomotor activity (wheel revolutions) prior to (Water) and during Meth treatment (Meth, final 2 weeks of treatment). Mean ± SEM. n= 5-8. Two-way ANOVA, uncorrected Fisher's LSD test; ns, not significant; $*P<0.05$; $***P<0.001$.

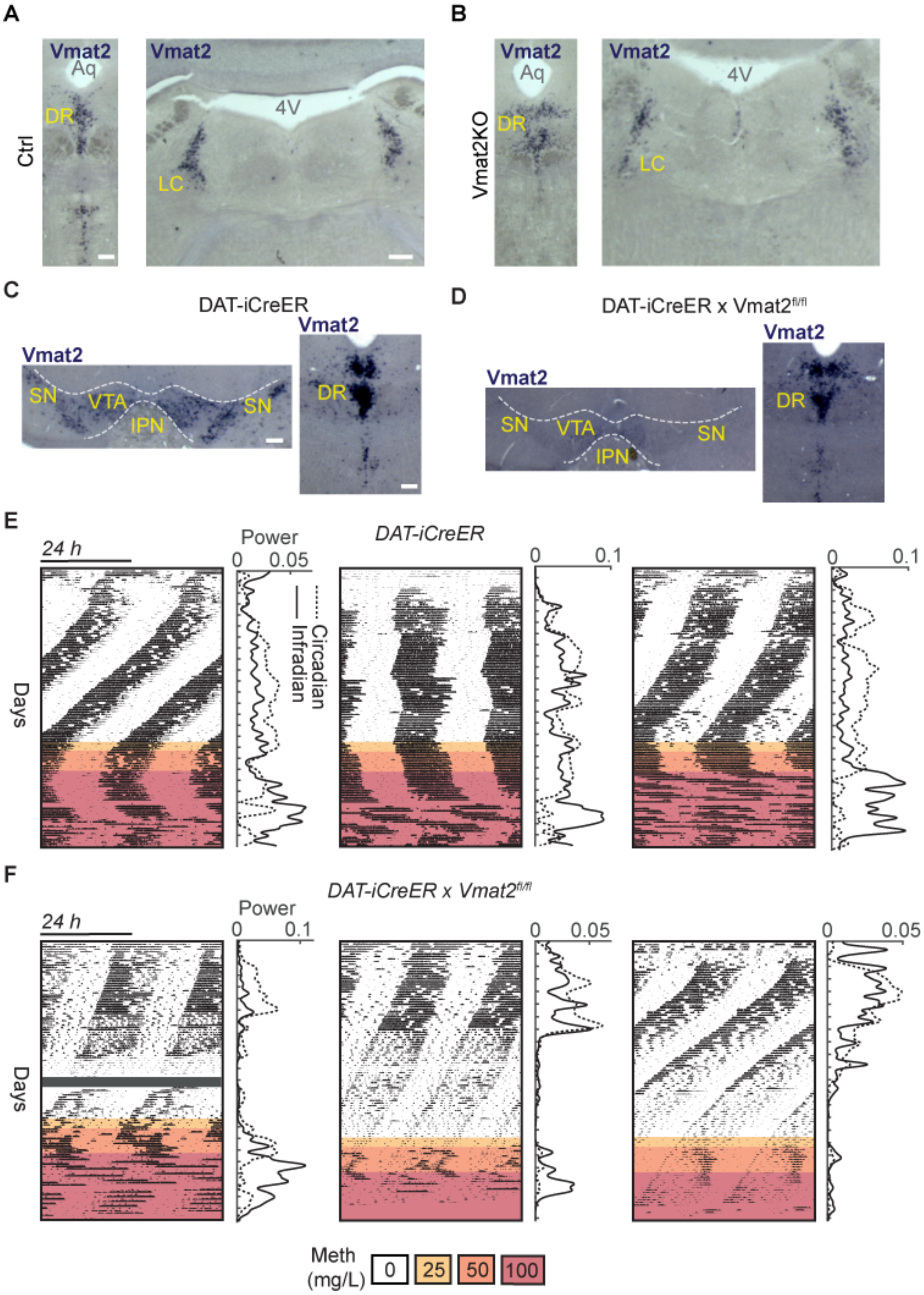

fig. S6. *Vmat2* gene disruption and infradian rhythm generation. (**A, B**) In situ hybridization with a *Vmat2* riboprobe showing preservation of *Vmat2* signal in raphe nucleus (left) and locus coeruleus (right) in ^{VTA}Vmat2KO mice (B) versus controls (A). Scale bars, 500 µm (LC), and 100 µm (Raphe). DR dorsal raphe; LC, locus coeruleus. (**C, D**) In situ *Vmat2* expression analysis of *DAT-iCreER* (C) and *DAT-iCreER*

x Vmat2$^{fl/fl}$ (D) mice upon tamoxifen treatment revealing selective loss of *Vmat2* transcripts in the midbrain (D, left) but not raphe nucleus (D, right) in *DAT-iCreER x Vmat2$^{fl/fl}$* mice compared to controls (C). Scale bars, 250 µm (VTA), and 100 µm (Raphe). (**E, F**) Adult *DAT-iCreER* and *DAT-CreER x Vmat2$^{fl/fl}$* mice were tamoxifen-injected daily for 5 days starting on day 4 of the running wheel recording to produce ^{DAT}Vmat2KO and respective control animals. Traces along-side the actograms show the scaled-average continuous wavelet transform spectrum in the circadian (20-27 hr) and infradian (27-96 hr) range. ^{DAT}Vmat2KO mice (F) but not controls (E) showed a gradual reduction in locomotor activity. Addition of Meth (indicated by orange background hues) to the drinking water rescued activity and led to the emergence of infradian components in both controls and ^{DAT}Vmat2KO animals as evidenced by the increased spectral power in the infradian range (solid trace).

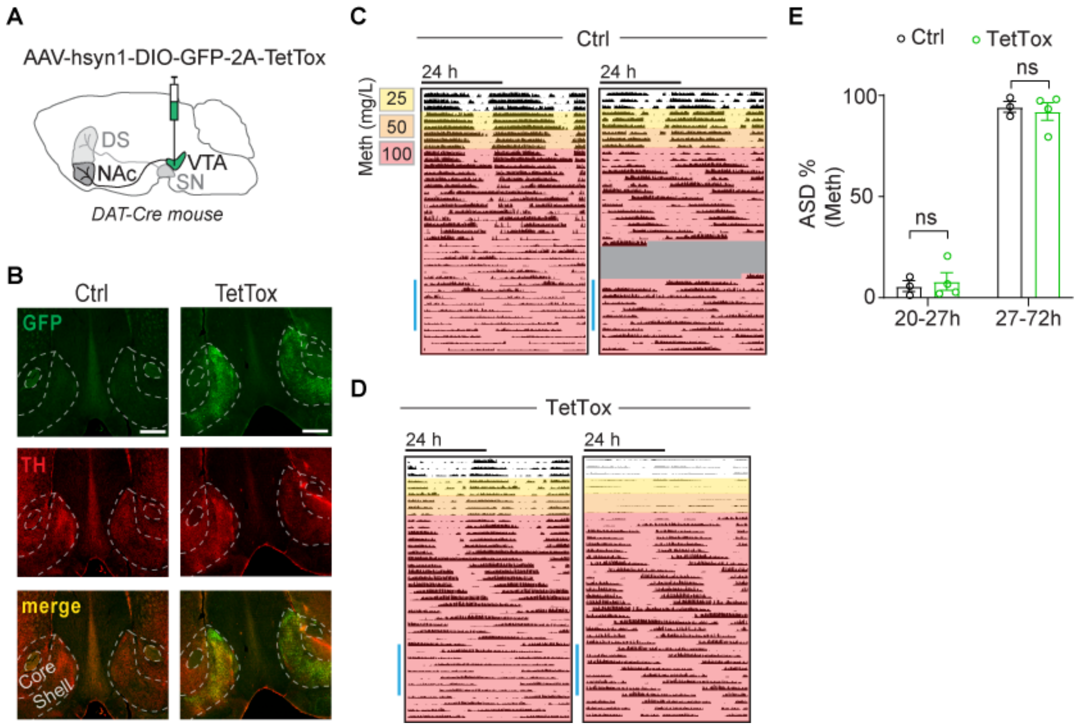

fig. S7. Tetanus toxin-mediated silencing of ^{VTA}DA neurons does not abrogate the 2ndC induction capacity. (**A**) AAV-hSYN1-DIO-GFP-2A-TetTox was bilaterally injected into the VTA of *DAT-Cre* mice. (B) Immuno-labeling for TH and GFP demonstrates effective transduction of NAc-shell projecting TH+ fibers. Scale bars, 500 µm. (**C, D**) Representative actograms of (C) Ctrl (top) vs (D) ^{VTA}TetTox (bottom) mice showing running wheel activity in response to Meth-supplemented drinking water indicated by color shading. Greyed area, data loss. (**E**) ASD analysis of periodograms derived from the time span indicated by blue bar next to actograms. Infradian rhythmicity under Meth was indifferent between ^{VTA}TetTox mice and controls, suggesting that ^{VTA}DA neuronal silencing does not abrogate the 2ndC emergence capacity. Mean ± SEM. n= 3-4. Two-way ANOVA with Bonferroni's multiple comparison. ns, not significant.

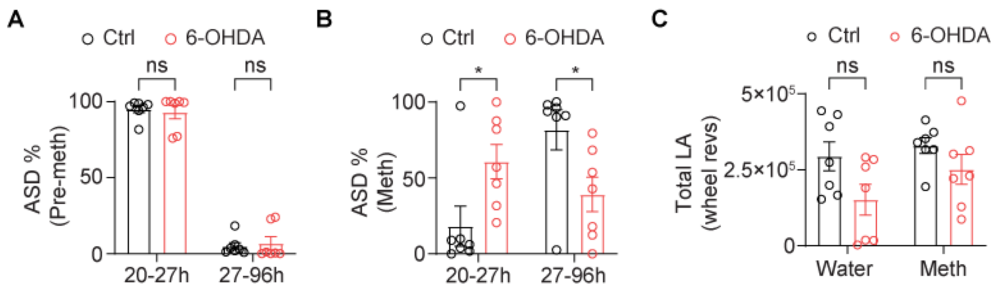

fig. S8. Loss of infradian rhythm generation capacity upon ablation of DA projections in the NAc. (**A, B**) Percent of significant rhythmic periodicities in the circadian (20-27 hr) and infradian (27-96 hr) period range before (A) and during the final 2 weeks of Meth-exposure (B). ASD, amplitude spectral density. (**C**) Total locomotor activity (wheel revolutions) of 6-OHDA and Ctrl mice prior to (Water) and during Meth treatment (Meth). Mean ±SEM. n= 7. Two-way ANOVA with Bonferroni's multiple comparison. ns, not significant; *P<0.05.

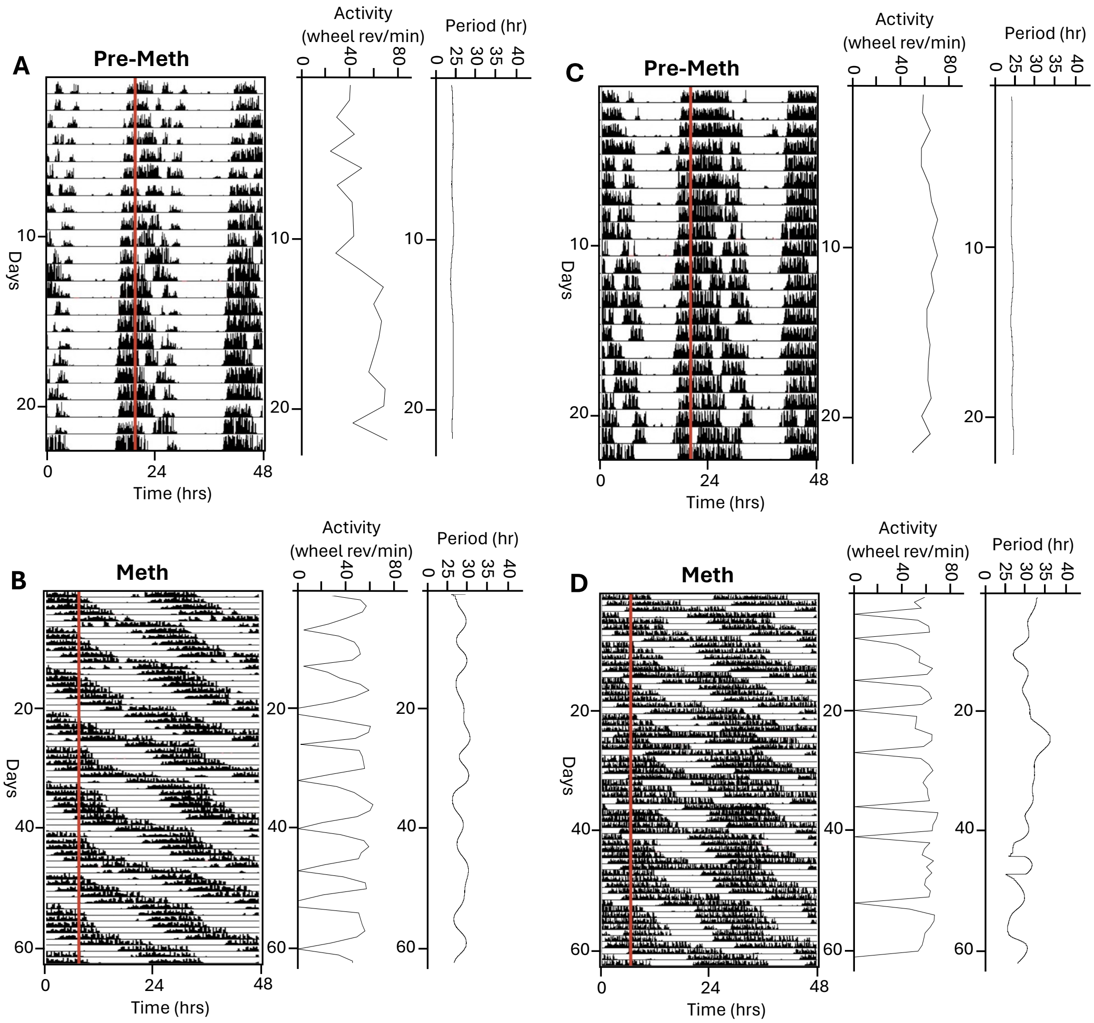

fig. S9. Infradian rhythmic mice showing evidence of beat cycling with regard to daily locomotor activity. Running wheel activity in constant darkness of mice that reached infradian rhythmicity at periods <48hr. Shown are the respective actograms prior (**A**, **C**) and during (**B**, **D**) treatment with 100mg/l Meth in drinking water. Middle: mean wheel revolutions per minute during a 2-hour window centered at the mean peak of daily activity indicated by the red vertical line in the actogram. Right: Instantaneous peak period of locomotor rhythmicity based on continuous wavelet transform analysis.
